# Room Temperature Broadband Bi_2_Te_3_/PbS Colloidal Quantum Dots Infrared Photodetectors

**DOI:** 10.3390/s23094328

**Published:** 2023-04-27

**Authors:** Lijing Yu, Pin Tian, Libin Tang, Wenbin Zuo, Hefu Zhong, Qun Hao, Kar Seng Teng, Guiqin Zhao, Runhong Su, Xiaoxia Gong, Jun Yuan

**Affiliations:** 1 School of Optics and Photonics, Beijing Institute of Technology, Beijing 100081, China; 2 Kunming Institute of Physics, Kunming 650223, China; 3 Yunnan Key Laboratory of Advanced Photoelectronic Materials & Devices, Kunming 650223, China; 4 School of Materials and Energy, Yunnan University, Kunming 650500, China; 5 Department of Electronic and Electrical Engineering, Swansea University, Bay Campus, Fabian Way, Swansea SA1 8EN, UK

**Keywords:** photodetector, room temperature, colloidal quantum dots (CQDs), Bi_2_Te_3_, synergy effect, broadband

## Abstract

Lead sulfide colloidal quantum dots (PbS CQDs) are promising optoelectronic materials due to their unique properties, such as tunable band gap and strong absorption, which are of immense interest for application in photodetectors and solar cells. However, the tunable band gap of PbS CQDs would only cover visible short-wave infrared; the ability to detect longer wavelengths, such as mid- and long-wave infrared, is limited because they are restricted by the band gap of the bulk material. In this paper, a novel photodetector based on the synergistic effect of PbS CQDs and bismuth telluride (Bi_2_Te_3_) was developed for the detection of a mid-wave infrared band at room temperature. The device demonstrated good performance in the visible-near infrared band (i.e., between 660 and 850 nm) with detectivity of 1.6 × 10^10^ Jones at room temperature. It also exhibited photoelectric response in the mid-wave infrared band (i.e., between 4.6 and 5.1 μm). The facile fabrication process and excellent performance (with a response of up to 5.1 μm) of the hybrid Bi_2_Te_3_/PbS CQDS photodetector are highly attractive for many important applications that require high sensitivity and broadband light detection.

## 1. Introduction

Infrared photoelectric detectors are widely used in the field of communication, surveillance, night vision, and biomedical [1]. There has been a continuous demand for high-performance infrared photodetectors. For example, there are increasing demands for low-cost, low-power, large-array photodetectors that can demonstrate multi-color detection with high sensitivity. Mercury cadmium telluride (HgCdTe) is currently the most commonly used material in infrared photodetectors because of its adjustable band gap [2]. However, its application is limited due to many disadvantages, such as high growth cost, complex device fabrication process, refrigeration requirement, and mismatch with silicon lattice. To integrate HgCdTe on a silicon readout circuit, a flip interconnection process is used. First, HgCdTe is segmented according to the requirements of the photodetector array by photolithography. Indium bumps are then grown on each pixel, which are mechanically bonded to the silicon readout circuit by indium. The reliability of the entire bonding and interconnection process depends on the size and alignment accuracy of the HgCdTe and the silicon readout circuit, especially when the pixel spacing (e.g., the center distance between adjacent pixels) becomes smaller; hence accurate alignment becomes more difficult. Inherent difficulties in interconnection, such as misalignment and unreliable connections, can lead to pixel defects and low yields, which increase the cost of producing high-resolution arrays [3].

There have been many research activities to develop new materials that can be used in the development of low-cost, high-performance infrared photodetectors which can operate at high temperatures. The use of quantum dots in optoelectronic devices has attracted much attention in recent years due to their unique properties, including surface effect [4], quantum confinement effect [5], quantum tunneling effect [6], dielectric confinement effect [7], and coulomb blockade effect [8]. For example, the band gap of colloidal quantum dots and its spectral response range can be tuned by controlling the size of the quantum dots due to the quantum confinement effect [9]. The size of the dots can be controlled by changing the precursor concentration, reaction temperature, and time during the synthesis process.

In recent years, researchers have developed a variety of synthesis and preparation techniques that provide a well-controlled size and shape of the synthesized quantum dots. In order to improve the quality and stability of the quantum dots, different synthesis media, chemical precursors, and ligands have been developed. Synthesis temperature and passivation ligands are the key factors affecting the synthesis of quantum dots. Temperature provides energy to overcome the activation barrier of quantum dot growth, and ligands promote the growth of quantum dots by modifying the growth environment. It is important to select appropriate ligands as they can directly affect the solubility and supply of active components during the synthesis process of the quantum dots and can reduce its surface energy hence allowing them to reach a stable state, as well as improving the functionality of the quantum dots [10].

Furthermore, the nanomaterial can be processed in solution, which is conducive to the large-scale manufacturing of quantum dots-based devices on wafers [3], and is considered an ideal material for use in the next generation of photodetectors [11]. Currently, quantum dots have been widely used in photodetectors [12], solar cells [13], and light displays [14]. Due to the inherently high surface-to-volume ratio of quantum dots, the surface properties of the quantum dots can significantly impact their physical properties, including photophysics, charge transport, catalysis, etc. The photoelectric properties of quantum dots can be engineered by chemically modifying their surface through oxidation or ligand exchange [15]. Surface ligands play an important role in the synthesis of quantum dots as they control the nucleation, growth, and stability of the quantum dots. The surface ligands also play a vital role in assembling individual quantum dots into a thin film. They determine the spacing and interaction between the quantum dots, and the spacing has an important effect on the electronic properties of the quantum dot thin film. For example, the surface ligands can directly affect the conductivity and energy structure of quantum dots. Brown et al. reported the influence of different surface ligands on the positions of the Fermi level (E_F_), conduction band (E_CB_), and valence band (E_VB_) of quantum dots [16].

As colloidal quantum dots can be synthesized and processed in solution, a variety of low-cost deposition techniques can be used to prepare quantum dot thin films [17]. For example, these quantum dots can be coated, printed, or deposited onto a small pixel pitch of a high-resolution silicon readout circuit, hence circumventing the flip interconnection process. This would allow facile large-scale manufacturing as well as improve the infrared focal plane array to diffraction-limited pixel pitch in order to achieve compact and high-definition imaging. In addition, the coating process of quantum dots will enable wafer-level fabrication, which will significantly reduce the production cost of quantum dot infrared focal plane arrays [3]. PbS CQDs are one of the most widely studied quantum dot materials. The band gap of PbS CQDs can be tuned by adjusting their size and is often used as the active layer of infrared photodetectors because of their high absorption coefficient. However, there is difficulty in collecting photogenerated carriers as they usually recombine with opposite carriers or capture by “traps” prior to the collection due to the low mobility of the carriers (10^−5^–10^−2^ cm^2^V^−1^s^−1^) [18], hence limiting the photoelectric performance of the device. In order to improve the charge transport and carrier mobility in PbS CQDs, a short-chain ligand has been employed in linking the dots instead of using long-chain ligands and has shown an enhancement in the electronic coupling between the quantum dots [19]. The performance of quantum dot devices can also be effectively improved by combining quantum dots with other materials. Guo et al. reported the hybridization of PbS quantum dots with high-quality single-layer graphene grown by CVD. They studied the photoinduced charge transfer reaction between PbS quantum dots and graphene and analyzed the internal mechanisms of charge injection, regeneration, and recombination at the interface of graphene and quantum dots, which effectively improved carrier mobility [20]. Several studies on the hybrid structures of quantum dots with other materials have been reported. Quantum dots, which exhibit good solubility, can easily combine with other materials, including two-dimensional materials (e.g., graphene [21], black phosphorouss [22], MXene [23], TMDs [22], Bi_2_O_2_Se [24], etc.), bulk materials (e.g., GaAs) [25], and other quantum dot materials [26]. These hybrid structures can achieve high carrier mobility and optical absorption at the same time through the synergistic effect between quantum dots and other materials [27]. Yilin Sun et al. [23] reported a hybrid 2D/0D photodetector using 2D metal MXene as an electrode of the photodetector and 0D PbS quantum dots as a photoactive layer. The photodetector exhibited broadband optical response from visible to near-infrared band with high detectivity of up to 2.4 × 10^11^ Jones. It is worth noting that photodetectors consisting of hybrid quantum dots with other materials operate mainly in the UV-near infrared band, with limited expansion beyond the band.

The quantum confinement effect can increase the optical bandgap of semiconducting CQDs from a minimum value, which is defined by the bandgap of the respective bulk materials. In other words, for CQDs to generate photoelectrons in mid- and long-wave infrared (MWIR and LWIR, respectively), the bandgap of the bulk semiconductor material has to be very small (<0.4 eV). As the bandgap of bulk PbS is ~0.41 eV [26], the potential application of PbS quantum dots for photodetection in MWIR and LWIR bands is, therefore, very limited. In order to overcome this limitation, other researchers have investigated the method of “heavy-doping” to develop a PbS CQDs-based photodetector for mid-wave detection. Ramiro et al. [28] reported intraband absorption and photoelectric detection in the range of 5–9 μm via heavy doping of PbS colloid quantum dots. The photodetector demonstrated a responsivity of ~10^−4^ A/W at 80 K. The intraband photodetection in the MWIR and LWIR range (e.g., 5–9 μm) exceeded the bulk band gap of PbS, hence overcoming the limitation of the bulk band gap in optoelectronics. However, such a method cannot produce stable, heavily doped solid films and requires a complicated process. At present, the electronic doping of lead chalcogenide nanocrystals remains a challenge.

In this work, a novel broadband photodetector based on the combination of PbS CQDs and Bi_2_Te_3_ is developed and studied. Bi_2_Te_3_ has a narrow bulk bandgap (~0.15 eV), strong light absorption characteristics, and ultra-high carrier mobility, which is highly suitable for mid-long wave infrared light detection at room temperature [29]. The combination of PbS CQD and Bi_2_Te_3_ shows an effective improvement in device performance [30]. When compared with the PbS CQDs photodetector, the hybrid Bi_2_Te_3_/PbS CQDs photodetector exhibits good performance in the Vis-NIR band and also demonstrates spectral response in the MWIR band (between 4.6 and 5.1 nm). Importantly, the facile preparation method of the Bi_2_Te_3_/PbS CQDs photodetector, as compared with the heavy-doping method for mid-wave detection, is attractive for extensive applications. The mechanism for the observed high-performance broadband (between visible and mid-wave infrared light) response of the Bi_2_Te_3_/PbS CQDs photodetector at room temperature is discussed in this paper.

## 2. Materials and Methods

### 2.1. Materials

The materials used in the experiments were PbS CQDs, n-octane, tetrabutyl-ammonium iodide (TBAI), methanol, NH_3_·H_2_O, H_2_O_2_, Bi_2_Te_3_ target material (99.99%), and Al (99.99%). Unless otherwise stated, all chemicals were commercially purchased and used without further purification. N-octane was purchased from Tianjin Zhiyuan Chemical Reagent Co., Ltd. (Tianjin, China), TBAI was purchased from Shanghai Titan Scientific Co., Ltd. Methanol, NH_3_·H_2_O, and H_2_O_2_ were purchased from Tianjin Fengchuan Chemical Reagent Co., Ltd. (Shanghai, China). Bi_2_Te_3_ target (99.99%) and Al (99.99%) were purchased from Zhongnuo Advanced Materials Technology Co., Ltd. (Beijing, China). PbS CQDs coated with oleic acid were synthesized by the cation-exchange synthesis method [31]. The prepared PbS CQDs were dissolved in n-octane with a concentration of 30 mg/mL and stored in a glove box filled with nitrogen.

### 2.2. Device Fabrication

First, the quartz substrate was cleaned in a mixed solution (NH_3_·H_2_O:H_2_O_2_:H_2_O = 1:1:3) in a chemical bath at 80 °C for 30 min and then blow-dried with nitrogen gas for later use. A film of Bi_2_Te_3_ was sputtered on the cleaned quartz substrate at a power of 200 W, pressure of 5 Pa, and sputtering time of 1 s with constant Ar flow at 60 standard cubic centimeters per minute (sccm). The sputtered Bi_2_Te_3_ film was rapidly annealed in a vacuum oven at 300 °C for 10 min. The annealed Bi_2_Te_3_ film was then spin-coated with PbS CQDs. A total of 50 μL PbS CQDs solution was spun on the Bie_2_Te_3_ film at 2500 revolutions per minute (rpm) for 30 s. After spin-coating a layer of PbS CQDs, TBAI ligand exchange was carried out, which consisted of drop-casting a 100 μL TBAI solution onto the PbS CQDs film and allowing 60 s for the reaction to take place before spin-coating at 2500 rpm for 30 s, and then the swapped long-chain ligand was washed off by rotary washing with methanol for three times. Eight layers of PbS CQDs films were prepared by repeating the above processes. Finally, aluminum electrodes with a thickness of 95 nm were evaporated onto the surface of PbS CQDs films with a shadow mask using a customized physical vapor deposition (PVD) device (at a pressure of 7.0 × 10^−5^ Pa). The effective photosensitive area of the device was 3.0 mm^2^.

### 2.3. Characterization

A variety of characterization techniques were used to investigate the properties of PbS quantum dots and Bi_2_Te_3_ materials. Transmission electron microscopy (TEM) (Tecnai G2-TF30, FEI Inc., Hillsboro, OR, USA) was used to analyze the shape, size, size distribution, and electron diffraction patterns of the materials. An ultraviolet-visible spectrophotometer (UV-VIS-NIR 3600, Shimadzu Inc., Kyoto, Japan) was used to study the optical properties of the materials. Atomic force microscopy (AFM) (Seiko SPA-400, Hitachi Inc., Tokyo, Japan) was used to study the thickness and surface morphology of the materials. The structure, vibration and rotation modes of the materials were studied using Raman spectroscopy (Renishaw inVia, Renishaw Inc., York, UK).

Current and voltage (*I-V*) characterizations of the device under dark conditions and light illumination using LED with different wavelengths as incident light sources were performed in a dark box to analyze the performance of the device. A signal generator (DG1022U, RIGOL Technology Co., Ltd, Beijing, China) was used to drive the LED in order to study the transient response of the device. Measurements on the device were performed using a digital source meter (Keithley 2400, Tektronix Inc., Beaverton, OH, USA). A silicon light power meter (FZ400, China Education Au-Light Technology Co., Ltd., Beijing, China) was used to calibrate the optical power density of the LED light source.

## 3. Results and Discussion

**Figure 1:**
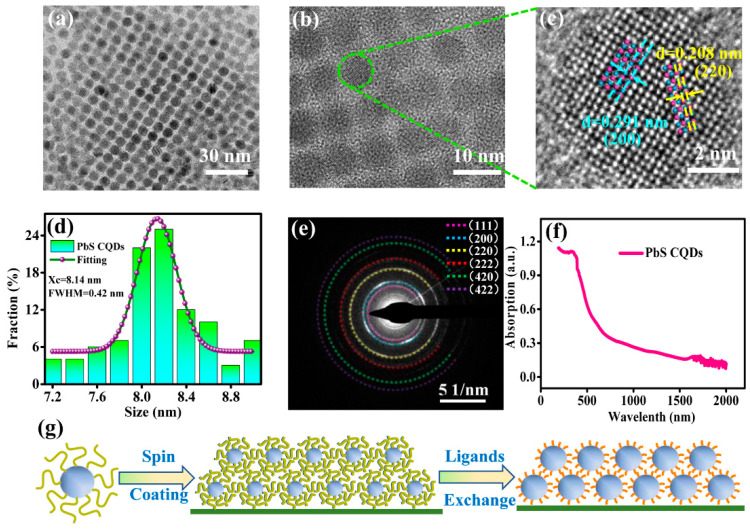
(**a**,**b**) TEM images of PbS CQDs. (**c**) HRTEM image of PbS CQDs. (**d**) PbS CQDs size distribution histogram. (**e**) FFT image of PbS CQDs. (**f**) Optical absorption spectrum of PbS CQDs. (**g**) Schematic diagram illustrating the ligand exchange process of PbS CQDs.

PbS CQDs film is used as a photosensitive layer in the photodetector; hence its surface morphology can affect the electrical conductivity of the film, which can have a direct impact on the performance of the device. Therefore, it is important to obtain dense and uniform photosensitive film to ensure the good performance of the device. Particularly, uniformity in the size and shape of CQDs is necessary to achieve high-quality multilayers of CQDs film [32]. Transmission electron microscopy (TEM) images of PbS CQDs are shown in Figure 1a,b. As shown in Figure 1a, the synthesized PbS CQDs self-assemble into an ordered superlattice. Figure 1b shows that each quantum dot exhibits good crystallinity. A high-resolution (HR) TEM image of CQDs is shown in Figure 1c for further analysis of the crystallinity and crystal structure of PbS CQDs. The lattice fringes of (220) and (200) crystal planes, as shown in the HRTEM image, are in good agreement with PbS CQDs crystal structures. Figure 1d shows the size distribution histogram of the PbS CQDs in Figure 1a. It can be seen that the average size of the PbS CQDs is 8.14 nm, and the full width at half maximum (FWHM) is 0.42 nm, indicating that the CQDs in this work exhibit good size uniformity and mono-dispersity compared with CQDs synthesized by other methods [33]. The fast Fourier-transform (FFT) image, which reveals the crystal structure of the PbS CQDs, is shown in Figure 1e. Figure 1f shows the absorption spectrum of the PbS CQDs, and an exciton absorption peak at 1700 nm is evident. The band gap energy of PbS CQDs can be calculated using the following formula:

(1) $$E_{g}=\frac{1240}{\lambda }\;\;\;\;$$

where *E_g_* is the band gap energy, and λ is the wavelength corresponding to the first exciton absorption peak. The calculated band gap energy of PbS CQDs with an exciton absorption peak at 1700 nm is 0.73 eV. The physical and chemical properties of PbS CQDs are not affected after they were coated onto the Bi_2_Te_3_ film; hence its band gap remains at 0.73 eV.

The stability of quantum dots can be affected by synthesis methods and stabilizing agents [34,35]. The first exciton absorption peak of 1700 nm was used in this experiment on relatively large-size quantum dots. The cation exchange method was used to synthesize the quantum dots instead of the traditional thermal injection synthesis methods because it is more conducive to producing stable quantum dots due to in situ chloride passivation [31].

The surface ligand and ligand exchange mechanism in CQDs play a crucial role in the film formation. After the PbS CQDs were synthesized, the surface was coated with long-chain organic ligands, which assist in obtaining monodisperse CQDs during the synthesis process and also maintaining the solubility of the CQDs in different solvents. The presence of long-chain ligands significantly reduces the overlapping of wave functions between neighboring CQDs, hence inevitably forming a potential barrier and inhibiting the electron transport between CQDs [36]. The layer-by-layer (LBL) exchange method was used for the ligand exchange. After ligand exchange, the volume previously occupied by long-chain ligands usually leads to gaps or “cracks”. In order to circumvent the effect of these gaps or “cracks”, spin coating of a multilayer of CQDs is needed. Figure 1g illustrates the ligand exchange process of the PbS CQDs.

**Figure 2:**
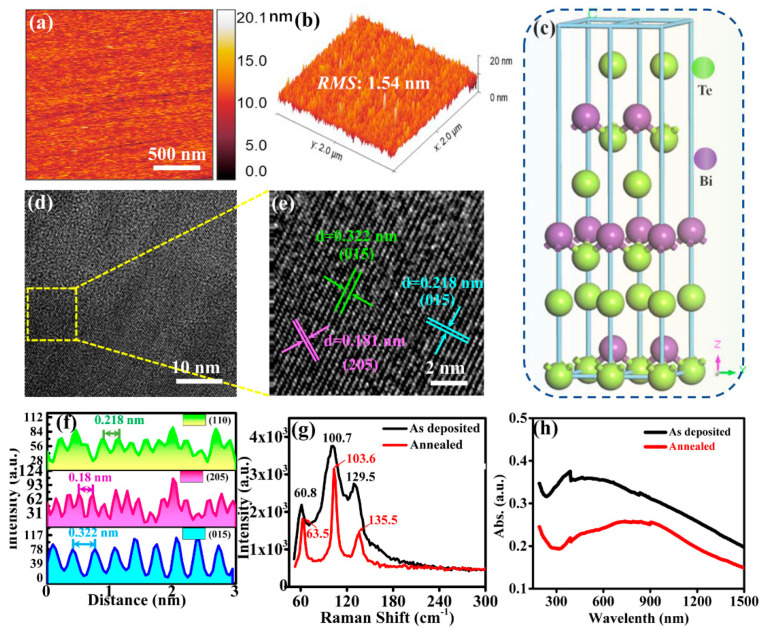
(**a**) AFM image on Bi_2_Te_3_ surface. (**b**) 3D profile of Bi_2_Te_3_ surface obtained from AFM. (**c**) Schematic diagram depicting the crystal structure of Bi_2_Te_3_. (**d**) TEM image of Bi_2_Te_3_ film. (**e**) HRTEM image of Bi_2_Te_3_ film. (**f**) Line profiles showing the interplanar spacings of different crystal planes in (**e**). (**g**) Raman spectra of Bi_2_Te_3_ film before and after annealing. (**h**) Optical absorption spectra of Bi_2_Te_3_ film before and after annealing.

Atomic force microscopy (AFM) images of annealed Bi_2_Te_3_ film used in the device are shown in Figure 2a,b. The root mean square (RMS) of the film is 1.54 nm, indicating that the prepared Bi_2_Te_3_ film (with a thickness of ~7 nm) has a relatively smooth surface, which is essential for the high-quality formation of PbS CQDs film later. Figure 2c depicts the lattice structure of Bi_2_Te_3_. Bi_2_Te_3_ is a rhomboid structure belonging to the R-3m space group (*a* = *b* = 4.386 Å, *c* = 30.497 Å, and α = β = 90°, γ = 120°). The layered crystal structure of Bi_2_Te_3_ composes of five atomic layers Te(1) -Bi-Te (2) -Bi-Te (1). Therefore, the Bi atom in Bi_2_Te_3_ has three adjacent Te(1) atoms. The length of the nearest Te(1)-Bi bond is 3.065 Å, and the length of the three Te(2)-Bi bonds is 3.246 Å [37]. The quintuple atomic layers (QLs) are bonded by strong covalent bonds, while the bonding between the QLs is via weak van der Waals forces [38]. The TEM image, shown in Figure 2d, reveals good crystallinity of Bi_2_Te_3_ after annealing. From the HRTEM image of Bi_2_Te_3_ in Figure 2e, the lattice spacings are 0.322, 0.181, and 0.218 nm, corresponding to the (110), (205), and (015) crystal planes of Bi_2_Te_3_, respectively. The line profiles of these lattice strips are shown in Figure 2f. Figure 2g shows the Raman spectrum of Bi_2_Te_3_ before and after annealing. Before the annealing of Bi_2_Te_3_, its characteristic peaks are situated at 60.8, 100.7, and 129.5 cm^−1^. After annealing, these peaks red shift to 63.5, 103.6, and 135.5 cm^−1^, which suggests a change in the crystal structure of the film after annealing. Furthermore, these peaks are narrower, hence indicating that the crystallinity of Bi_2_Te_3_ has enhanced after annealing. The absorption spectra of Bi_2_Te_3_ before and after annealing are shown in Figure 2h. It is evident that the absorption spectrum of Bi_2_Te_3_ after annealing has red shifted, which is consistent with the Raman results. The vibration modes that can be detected by Raman are E_g_, A_1g_, E_u,_ and A_1u_ [39]. The peaks observed in Figure 2g correspond to A^1^_1g_ (60.8 cm^−1^), E^2^_g_ (100.7 cm^−1^), and A^2^_1g_ (129.5 cm^−1^), respectively, which are consistent with those reported in the literature [40].

**Figure 3:**
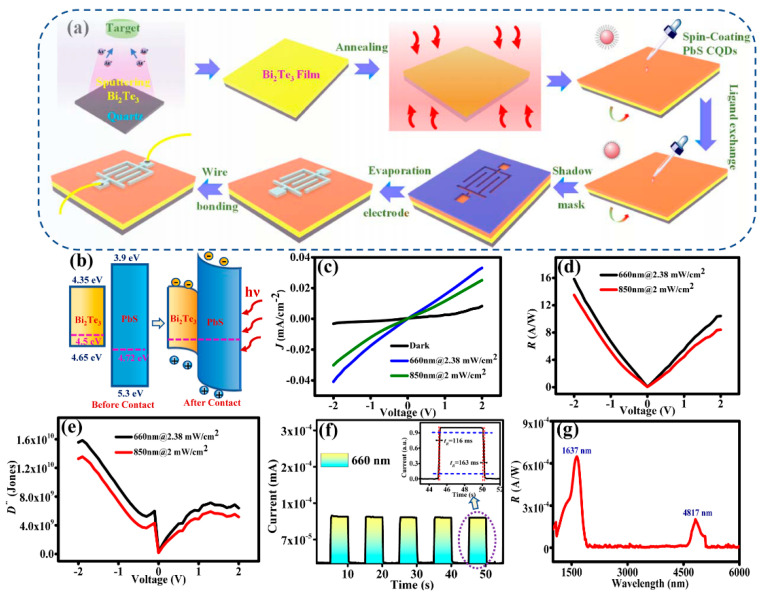
(**a**) Schematic diagrams illustrating the fabrication process of the Bi_2_Te_3_/PbS CQDs photodetector. (**b**) Band diagrams of the Bi_2_Te_3_/PbS CQDs heterostructure before and after contacting. (**c**) *I*-*V* curves of the photodetector under 660 and 850 nm illumination. (**d**) Plot of responsivity (*R*) against voltage (*V*). (**e**) Plot of detectivity (D*) against voltage (*V*). (**f**) Photocurrent switching behavior under 660 nm illumination. (**g**) Spectral response curve of the photodetector.

The fabrication process of the Bi_2_Te_3_/PbS CQDs photodetector is shown in Figure 3a. During sputtering, argon ions (Ar^+^) are accelerated and bombarded onto the Bi_2_Te_3_ target, resulting in the deposition of the particles onto the substrate to form a Bi_2_Te_3_ film. The crystalline phase of Bi_2_Te_3_ films grown under different conditions was characterized using XRD and XPS (results are shown in Discussion S1). As can be seen from Figure S1, materials grown at a sputtering power of 200 W exhibit the best crystallinity, and the gas rate has little effect on the material crystallinity, as shown in Figure S2. The sputtering power of 200 W and gas rate of 60 sccm were selected as the growth conditions. Figure S3 shows the XPS survey scan of Bi_2_Te_3_ films prepared under this condition. Bi and Te atomic ratio close to 2:3 is observed from the XPS spectrum.

After sputtering the Bi_2_Te_3_ film on the cleaned quartz substrate, it was then placed in a rapid annealing furnace for 10 min at 300 °C. A total of eight layers of PbS CQDs (with a thickness of ~120 nm) were coated on the annealed Bi_2_Te_3_ film by the LBL method and using TBAI for ligand exchange. Subsequently, aluminum interdigital electrodes, having an effective photosensitive area of 3.0 mm^2^, were deposited onto the prepared film. Finally, the electrodes were bonded with wire for device characterization. The thickness of Bi_2_Te_3_ and PbS QDs films has been optimized as the film thickness plays an important role in the performance of the device. Bi_2_Te_3_ is a topological insulator having metallic and insulating states at its surface and bulk, respectively. In order to obtain high carrier mobility at Bi_2_Te_3_ film, it is necessary to make the film as thin as possible so that a significant proportion of the material is in metallic states. Therefore, a film thickness of 7 nm was grown by magnetron sputtering for 1 s. As for PbS QDs film, a film thickness of about 100 nm is considered optimum. If the PbS QDs film is too thin, its absorption of light energy is limited. If it is too thick, the transmission of charge carriers will be affected.

The process of photoelectric conversion can be divided into the following two steps: (1) The photosensitive layer absorbs incident light to produce electron-hole pairs; (2) The extraction and transfer of photogenerated carriers by the electrode. Figure 3b shows the energy band diagram of the hybrid Bi_2_Te_3_/PbS CQDs structure and illustrates the physical mechanism of the photodetector. The Fermi level of Bi_2_Te_3_ is about 4.65 eV [41], and the Fermi level of PbS CQDs after TBAI ligand exchange is about 4.72 eV [16]. When the Bi_2_Te_3_ film and PbS CQDs come into contact, both Fermi levels align. The electrons of PbS CQDs would move to Bi_2_Te_3_, hence resulting in the accumulation of electrons in Bi_2_Te_3_ and holes in PbS CQDs. Once equilibrium is reached, a built-in electric field is formed between PbS CQDs and Bi_2_Te_3_ that causes the energy band of Bi_2_Te_3_ to bend downward and that of PbS CQDs to bend upward. When electron-hole pairs are generated during illumination, photogenerated carriers are rapidly separated in a carrier mean-free path and are swept away by the strong built-in electric field. Finally, these electrons and holes are collected by electrodes as a photocurrent externally. The hybrid Bi_2_Te_3_/PbS CQDs structure can effectively promote the transfer and extraction of charge carriers, which can improve the response speed of the device and therefore enhance the performance of the device.

The photoelectric detection performance of the Bi_2_Te_3_/PbS CQDs photodetector is studied. *I*-*V* characterization of the device in the visible band and spectral characterizations from near-infrared to mid-wave bands (e.g., 1–6 μm) were performed. Figure 3c shows the *I-V* characteristic curves of the device under 660 and 850 nm incident light and dark conditions. In order to evaluate the performance of the Bi_2_Te_3_/PbS CQDs photodetector, the responsivity (*R*) and detectivity (D*) of the device are determined using the following equations [25]:

(2) $$R=\frac{I_{L}-I_{D}}{A\times P_{opt}}$$

(3) $$D^{*}=\frac{A^{1/2}R}{{\left(2qI_{D}\right)}^{1/2}}$$

where *I_L_* is the current measured in the light, *I_D_* is the current measured in the dark, *P_opt_* is the incident light intensity, *q* is the unit charge, and *A* is the effective photosensitive area. The unit of D* is cm·Hz^1/2^/W, which is often written as Jones.

The *R* and D* at different wavelengths (660 and 850 nm) are obtained as shown in Figure 3d,e, respectively. The maximum values of *R* and D* at 660 nm are 16 mA/W and 1.6 × 10^10^ Jones, while the values at 850 nm are 13.5 mA/W and 1.35 × 10^10^ Jones, respectively. The transient response of the device is shown in Figure 3f; the rise and fall times of the device are 116 and 163 ms, respectively, as illustrated in the inset of Figure 3f. Figure 3g shows the spectral response of the device, which demonstrates a broadband response from visible light to mid-wave bands. The performance of the PbS device without Bi_2_Te_3_ is studied (as shown in Discussion S2), and the maximum values of *R* and D* at 660 nm of the device are 7.8 A/W and 4.4 × 10^9^ Jones, respectively. Between the two devices, the Bi_2_Te_3_/PbS hybrid device demonstrates much-improved performance.

However, the D* is not exceptionally high for PbS CQDs-based devices. This is probably due to a large density of defects in the PbS CQDs film, hence trapping a proportion of the photogenerated charge carriers. There is a need to improve the quality of PbS CQDs films in subsequent work. It is worth noting that there are two different active parts in the Bi_2_Te_3_/PbS CQDs photodetector, namely PbS CQDs and Bi_2_Te_3_. Since the incident light is directly incident on a relatively thick PbS CQDs film (~120 nm), the response from PbS CQDs is dominant. If the incident light outside the response range of PbS CQDs (such as the mid-wave band) manages to reach the Bi_2_Te_3_ layer, it would respond to the mid-wave band due to its much narrower band gap of 0.17 eV. As the incident light is partially absorbed by the PbS CQDs film before reaching a thin Bi_2_Te_3_ layer (~7 nm), only a small amount of light can reach the Bi_2_Te_3_ layer; hence the light response at the mid-wave band is relatively small. This explains the broadband response of the Bi_2_Te_3_/PbS CQDs photodetector, as shown in Figure 3g. The excellent broadband optical response of the device is therefore attributed to the effective optical absorption of PbS CQD, the wide-band response of Bi_2_Te_3_ material, and the synergistic effect between them.

## 4. Conclusions

In summary, a hybrid Bi_2_Te_3_/PbS CQDs photodetector for visible mid-wave infrared detection was produced and studied. The absorption band of Bi_2_Te_3_ film prepared by magnetron sputtering and post-annealing has extended to the mid-infrared band. After rapid thermal annealing, the Bi_2_Te_3_ film exhibits good crystallinity and a relatively smooth surface, which is important to develop high-quality spin-coated PbS CQDs films during device fabrication. The detection of MWIR, which is difficult to achieve using the PbS CQDs photodetector alone, is achieved by the hybrid Bi_2_Te_3_/PbS photodetector due to the synergistic effect of Bi_2_Te_3_ and PbS CQDs. In addition to responding to the MWIR band, the device also demonstrates excellent performance in the visible band. This work shows that the Bi_2_Te_3_/PbS CQD structure has great potential in the development of the next generation of broadband, high-performance, and low-cost infrared photodetectors.

## Data Availability

Not applicable.

## Supplementary Materials

The following supporting information can be downloaded at: https://www.mdpi.com/article/10.3390/s23094328/s1, S1. Discussion on magnetron sputtering process of Bi_2_Te_3_ film; S2. Discussion on PbS devices (without Bi_2_Te_3_).
