# Supplementary material for: Room Temperature Broadband Bi2Te3/PbS Colloidal Quantum Dots Infrared Photodetectors
Source: Sensors (Basel). 2023 Apr 27;23(9):4328. doi: 10.3390/s23094328 (PMC10181788; doi:10.3390/s23094328)
Supplement: Supplementary file 1 [file sensors-23-04328-s001.zip › sensors-2238710-supplementary.pdf]

Room Temperature Broadband Bi_2Te_3/PbS Colloidal Quantum Dots Infrared Photodetectors

Lijing Yu [1,2,3], Pin Tian [2,3], Libin Tang [1,2,3,*], Wenbin Zuo [2], Hefu Zhong [4], Qun Hao [1,*], Kar Seng Teng [5,*], Guiqin Zhao [2], Runhong Su [2], Xiaoxia Gong [2] and Jun Yuan [2]

1 School of Optics and Photonics, Beijing Institute of Technology, Beijing 100081, China
2 Kunming Institute of Physics, Kunming 650223, China
3 Yunnan Key Laboratory of Advanced Photoelectronic Materials & Devices, Kunming 650223, China
4 School of Materials and Energy, Yunnan University, Kunming 650500, China
5 Department of Electronic and Electrical Engineering, Swansea University, Bay Campus, Fabian Way, Swansea SA1 8EN, UK
* Correspondence: scitang@163.com (L.T.); qhao@bit.edu.cn (Q.H.); k.s.teng@swansea.ac.uk (K.S.T.)

S1. Discussion on magnetron sputtering process of Bi_2Te_3 film

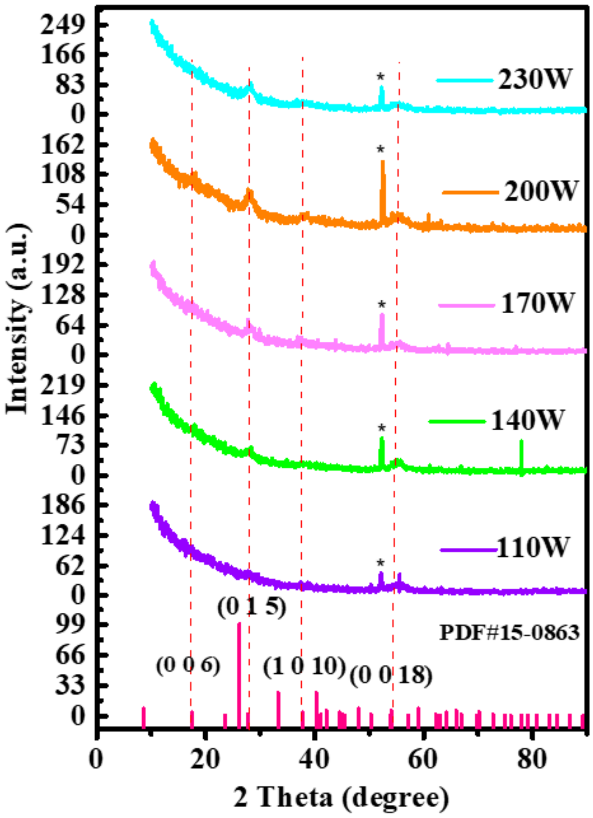

Figure S1. X-ray diffraction (XRD) patterns of Bi_2Te_3 film grown under different sputtering power

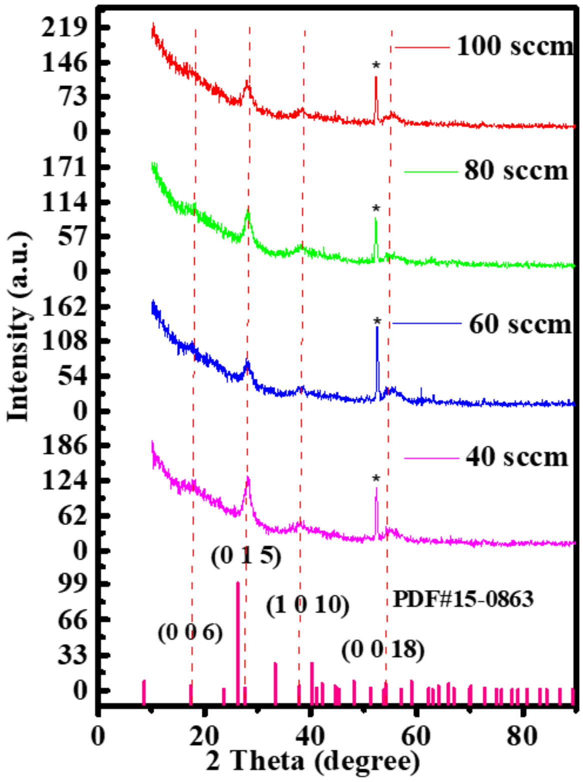

Figure S2. X-ray diffraction (XRD) patterns of Bi_2Te_3 film grown under different gas rate

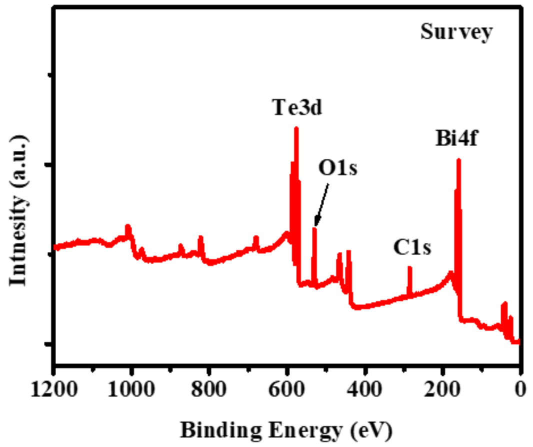

Figure S3. X-ray photoelectron spectroscopy (XPS) survey scan of Bi2Te_3 film

S2. Discussion on PbS devices (without Bi_2Te_3)

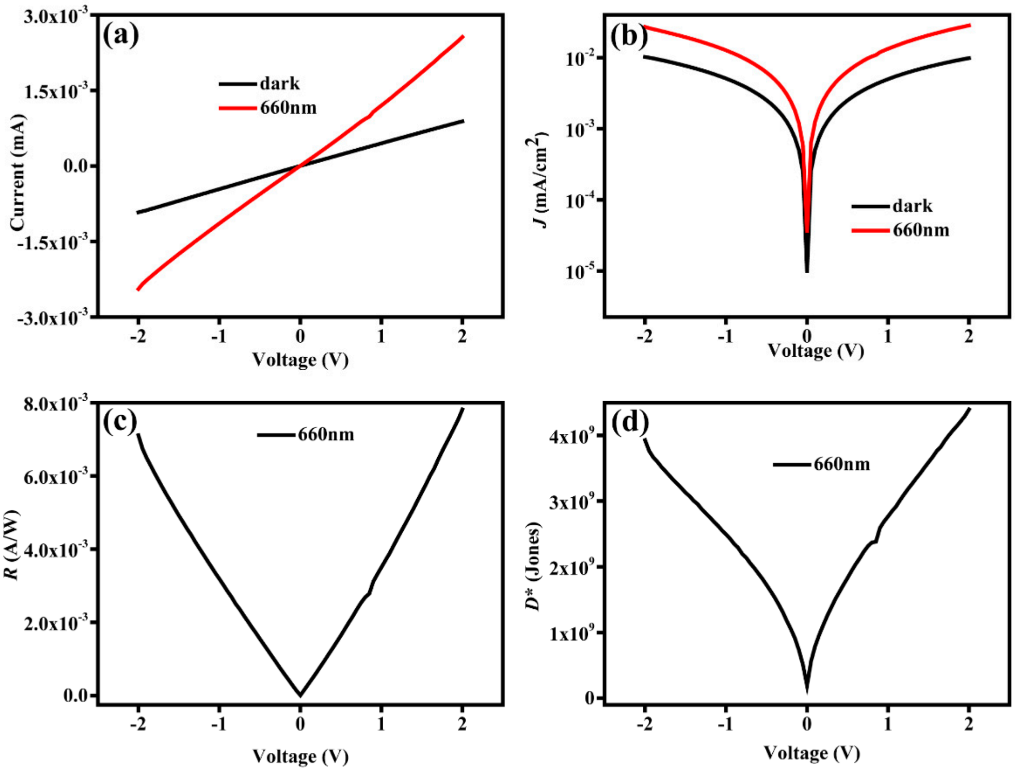

Figure S4. PbS devices without Bi_2Te_3. (**a, b**) *I-V* curves of the photodetector under 660 nm illumination. (**c**) Plot of responsivity (*R*) against voltage (*V*). (**d**) Plot of detectivity (*D**) against voltage (*V*).
